# Evidence of environmental niche differentiation in the striped mouse (*Rhabdomys* sp.): inference from its current distribution in southern Africa

**DOI:** 10.1002/ece3.219

**Published:** 2012-05

**Authors:** Christine N Meynard, Neville Pillay, Manon Perrigault, Pierre Caminade, Guila Ganem

**Affiliations:** 1 INRA, UMR CBGP (INRA/IRD/Cirad/Montpellier SupAgro), Campus international de BaillarguetCS 30016, F-34988 Montferrier-sur-Lez cedex, France; 2 School of Animal, Plant and Environmental Sciences, University of the WitwatersrandPrivate Bag 3, WITS 2050, South Africa; 3 Institut des Sciences de l’Evolution, UMR 5554, Université Montpellier 2C.C. 065, Montpellier cedex 5 34095, France

**Keywords:** MAXENT, niche differentiation, OMI, radiation, *Rhabdomys*, rodents, southern Africa, speciation, species distribution modelling

## Abstract

The aim of this study was to characterize environmental differentiation of lineages within *Rhabdomys* and provide hypotheses regarding potential areas of contact between them in the Southern African subregion, including the Republic of South Africa, Lesotho, and Namibia. Records of *Rhabdomys* taxa across the study region were compiled and georeferenced from the literature, museum records, and field expeditions. Presence records were summarized within a 10 × 10 km grid covering the study area. Environmental information regarding climate, topography, land use, and vegetation productivity was gathered at the same resolution. Multivariate statistics were used to characterize the current environmental niche and distribution of the whole genus as well as of three mitochondrial lineages known to occur in southern Africa. Distribution modeling was carried out using MAXENT in order to generate hypotheses regarding current distribution of each taxa and their potential contact zones. Results indicate that the two species within *Rhabdomys* appear to have differentiated across the precipitation/temperature gradient present in the region from east to west. *R. dilectus* occupies the wettest areas in eastern southern Africa, while *R. pumilio* occupies the warmer and drier regions in the west, but also penetrates in the more mesic central part of the region. We provide further evidence of environmental differentiation within two lineages of *R. dilectus*. Contact zones between lineages appear to occur in areas of strong environmental gradients and topographic complexity, such as the transition zones between major biomes and the escarpment area where a sharp altitudinal gradient separates coastal and plateau areas, but also within more homogeneous areas such as within grassland and savannah biomes. Our results indicate that *Rhabdomys* may be more specialized than previously thought when considering current knowledge regarding mitochondrial lineages. The genus appears to have differentiated along two major environmental axes in the study region, but results also suggest dispersal limitations and biological interactions having a role in limiting current distribution boundaries. Furthermore, the projection of the potential geographic distribution of the different lineages suggests several contact zones that may be interesting study fields for understanding the interplay between ecological and evolutionary processes during speciation.

## Introduction

The factors that determine species distributions have a long standing interest for ecologists (MacArthur 1982; Begon et al. 1996; Gaston 2003; Franklin 2009). Species distributions at large scales have traditionally been linked to climatic tolerances and broad-scale environmental conditions (MacArthur 1982; Guisan and Thuiller 2005). Variables such as mean and maximum temperatures have commonly been linked to physiological constraints that limit animal ranges at high latitudes or high altitudes (Graham et al. 2010; Rowe et al. 2010; Chen et al. 2011). Other commonly cited environmental variables include precipitation during the harshest months of the year, for example, the driest periods in desert habitats or the coldest periods in subpolar regions (Franklin 2009; Graham et al. 2010). Vegetation productivity, incorporated either through the use of satellite-derived vegetation indices or through an interaction between temperature and precipitation, has also been used frequently to model animal distributions because it can greatly affect resource availability and cover for mobile species (Buckley 2008; Franklin 2009). Finally, various environmental characteristics derived from altitude have been used to represent topographic hetero-geneity that may be an important limiting factor to dispersal and persistence in vertebrate populations (Franklin 2009; Rissler and Smith 2010; Rowe et al. 2010). Overall, the use of these multiple large-scale environmental predictors to project potential species distributions has proven highly successful in a variety of contexts (Graham et al. 2004; Guisan and Thuiller 2005; Elith and Leathwick 2009).

However, historic climate fluctuations are also known to influence the current geographical distribution of organisms, potentially leading to disjoint distribution patterns (Cruzan and Templeton 2000), reduced gene flow, and speciation because of diverging stochastic and selective pressures. Rodents rely on vegetation for food and cover, so by influencing the distribution of vegetation types, climate changes probably have had a major influence on their distribution (Frey 1992; Denys 1999). For example, Russo et al. (2010) explained the current patterns of diversification of the Namaqua rock mouse (*Micaelamys namaquensis*), a southern African rodent, through a series of arid periods associated with grassland expansions and savannas contractions during the Miocene and Pliocene (DeMenocal 2004). Drier periods fragmented species ranges into several small refuges, creating the opportunity for reproductive isolation and differentiation (Russo et al. 2010). Wetter periods allowed secondary contact between populations, generating the opportunity for either divergent selection or remixing of previously differentiated populations (Rundle and Nosil 2005; Rissler and Smith 2010). Similar explanations have been invoked to explain patterns of diversification among a variety of taxonomic groups and biogeographic regions such as the diversification of South American *Ctenomys* species (Mirol et al. 2010) and of South American birds (Fjeldsa et al. 1999; Herzog et al. 2005; Porzecanski and Cracraft 2005). Importantly, understanding current biogeographic patterns requires both taxonomic and range information to be available for a wide variety of taxa in a given geographic area. For example, the identification of suture zones, that is, areas in which several secondary contact zones overlap within a taxonomic group, has provided important evidence that mountain ranges can act as bio-diversity drivers in periods of climate change (Moritz et al. 2009; Rissler and Smith 2010). However, identifying such areas presupposes the existence of current distribution maps and taxonomic definitions for the taxonomic group under scrutiny.

**Figure 1:**
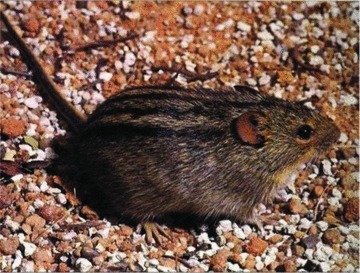
African striped mouse.

Many African rodents are believed to have radiated around 3 million years ago, possibly filling newly emerging habitats in a period of major environmental changes (Lecompte et al. 2008). Among them, many were traditionally considered as monospecific but were recently shown to display cryptic molecular divergence (Rambau et al. 2003; Nicolas 2006; Russo et al. 2010). However, the distribution ranges of these newly described lineages are often poorly known as well as their ecological characteristics and the mechanisms involved in their diversification. The African striped mouse (Fig. 1) belonging to the genus *Rhabdomys* exemplifies this problem. *Rhabdomys* belongs to the family Muridae, one of the most diverse rodent families for which southern Africa represents an important centre of endemism (Lecompte et al. 2008). Historically, *Rhabdomys* was regarded as monospecific, comprised only of the species *R. pumilio* (De Graaff 1981). However, recent mitochondrial DNA investigations have indicated the existence of two well-differentiated lineages leading the authors to propose two species names: *R. pumilio* described in the western (semi arid to arid) part of southern Africa, and *R. dilectus* described in the eastern part (mesic to humid) (Rambau et al. 2003). These authors also suggested further subdivisions within *R. dilectus*, with *R. d. dilectus* in the north and *R. d. chakae* in the south (Rambau et al. 2003). More recently, preliminary reports from ongoing studies have suggested even more subdivisions within the genus (Castiglia et al. 2011), indicating that the taxonomy of the genus needs revision. Moreover, laboratory crosses showing reduced fertility between these proposed species and subspecies (Pillay 2000) as well as evidence of diverging sexual recognition signals (Pillay et al. 2006) tends to validate these new taxonomic subdivisions.

Despite the fact that some differences in social systems and behavioural characteristics have been described within the genus (Schradin and Pillay 2005a; Pillay et al. 2006), environmental differentiation between the three taxa described above has not been previously formalized. The genus *Rhabdomys* is still described as a generalist clade because it occupies a variety of habitats, ranging from wet tropical areas to drier semiarid regions (Skinner and Chimimba 2005). However, the description of several lineages within *Rhabdomys*, and a better knowledge of their distribution create the possibility that the lineages might be specialized to different types of environments, and that the generalist label may therefore be questionable.

The specific aims of this study were to assess whether environmental differentiation parallels taxonomic diversification within *Rhabdomys* in southern Africa, to generate hypotheses regarding the potential distributions and contact zones between these taxa, and to discuss these results in the context of current climate and biogeography. We would expect the different taxa to differentiate along the major humidity and seasonality gradients present in southern Africa from east to west and from deserts to grasslands to savannas. Furthermore, based on dates of divergence provided in Rambau et al. (2003) and paleoclimatic information (DeMenocal 2004), we expect lineages of *R. pumilio* to have diverged in the arid western part of the southern African region and *R. dilectus* in the more humid eastern part of the southern African region. Here, we used multivariate statistics to relate the occurrence of lineages of genetically identified individuals with current environmental conditions. Furthermore, we produced spatial hypotheses of potential ranges for each lineage by using species distribution modeling in order to identify potential secondary contact zones and discuss these results in the context of past and present environmental conditions. Most importantly, this study allows for a first assessment of the potential environmental requirements of each taxon, and whether they are distinct enough to allow hypotheses on ecological radiation to be tested.

## Materials and Methods

### Study area

**Figure 2:**
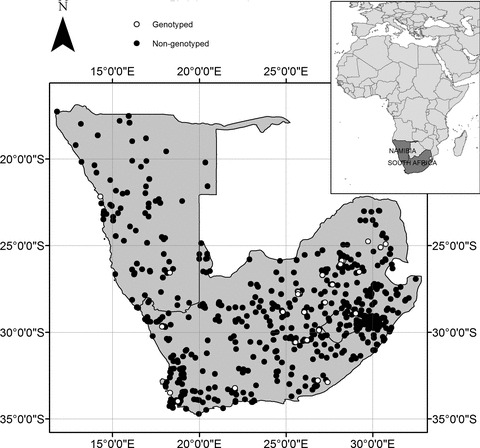
Geographic location of *Rhabdomys* occurrence records used in the analysis. Black dots indicate records of *Rhabdomys* obtained from various sources (see Appendix 1) whereas open circles indicate genotyped individuals.

Our study area comprised the southern African subregion including the Republic of South Africa, Namibia, and Lesotho (Fig. 2). This region includes several biomes in an environment mainly characterized by an east-to-west rainfall gradient, resulting in the arid, semiarid western region, and temperate grassland hinterland and coastal forests on the extreme southeastern coast. Most of the Republic of South Africa experiences summer rainfall, except for winter rainfall in the southwestern Cape region that has a Mediterranean climate, and the west coast that is semiarid. Temperature and precipitation vary according to geography, with the mountainous Drakensberg and Cape mountain regions experiencing lows of –6°C in winter and parts in the northern areas exceeding 40°C in summer (South African Weather Services: http://www.weathersa.co.za). Namibia is characterized mainly by desert and semidesert environments. The cold Benguela current along the coast creates fog but inhibits rainfall in the western regions. Rainfall is lower in the central plateau, and occurs during the summer months from November to February. There are two rainy seasons in the interior: a short rainy season between October and December and a longer rainy season from January to April. Summer temperatures range from 20°C to 40°C during the day, but drops sharply at night. Average minimum winter temperatures range between 6°C and 10°C, but can reach highs of between 18°C and 22°C (Namibia Meteorological Service; http://www.meteona.com). Finally, the Kingdom of Lesotho is a mountainous country, and comprises alpine and subalpine climate and vegetation. Temperature and precipitation are highly variable throughout the year. Winters are dry and cold, with mean minimum temperatures ranging from –6.3°C in the lowlands to 5.1°C in the highlands, although minimum temperatures can drop as low as –21°C. Snow is the main form of precipitation in winter. Summers are hot and humid, with mean maximum temperature ranging from 15°C to 32°C, but temperatures can drop to around 0°C. Thundershowers and hailstorms are common in summer, accompanied by strong winds. Annual precipitation ranges from 500 to 1200 mm, occurring mostly as rain during summer from October to April. Frost is common from late autumn to early spring (Lesotho Meteorological Services; http://www.lesmet.org.ls/climate_of_lesotho.htm). Overall, these three countries include the main distribution range of the genus *Rhabdomys* and also represent a wide range of environmental conditions.

### *Rhabdomys* records

Records of *Rhabdomys* were obtained from museum collections and published papers (Appendix 1). While some studies reported geographic coordinates resulting from a GPS (Global Positioning System) measurement, others (e.g., museum records) were referenced only by the trapping locality. In these cases, we obtained the geographic coordinates of those localities using Google Maps and different online geographic gazetteers. This resulted in 6083 records for our area of study. Considering the variable spatial error in this process (GPS measurements are usually more precise than a gazetteer search), we chose a large resolution of analysis that would likely absorb these errors. We divided the southern African subregion into an equal-area grid of 10 × 10 km. Any presence of *Rhabdomys* within a grid cell was recorded as a presence within a cell, whereas absence of any records was recorded as an absence. All environmental data were subsequently processed to reflect this resolution. Aggregating these individual records into present records at the 10-km grid resolution resulted in 520 presence records for all *Rhabdomys* taxa.

Among these records, 120 striped mice were assigned to a mitochondrial lineage based on published information of genotyped individuals (Rambau et al. 2003; Ganem et al. In Press). The numbers of presence records within the 10-km grid used in the analysis are shown in Table 1, and their spatial distribution in Figure 2.

**Table 1:** Number of grid cell records for the *Rhabdomys* lineages used in the statistical analysis. These correspond to the occurrences for *Rhabdomys* (*n*= 6083) and the genotyped individuals (*n*= 120, taken from Rambau et al. 2003 and Ganem et al. In Press) summarized within each 10 × 10 km grid cell as a presence (any number of records within the grid cell) or an absence (no records within that grid cell).

Clade	Number of occurrences
*Rhabdomys* sp.*	520
*Rhabdomys dilectus*	21
*Rhabdomys pumilio*	30
*Rhabdomys dilectus chakae*	12
*Rhabdomys dilectus dilectus*	10

* Includes all striped mice identified morphologically as belonging to the genus.

### Environmental data

Climate data and altitude were downloaded from the freely available Woldclim database (http://worldclim.org/). These data include climatic variables related to mean temperature and precipitation, as well as seasonality (see Appendix 1 for details), and were downloaded at a 30 arc-sec resolution (approximately 1 km). The data were then converted to a 10-km resolution by using ArcGIS 9.2 focal mean functions.

A vegetation productivity index, the Enhanced Vegetation Index (EVI), as well as a land cover layer were downloaded from the Modis Terra website (http://modis.gsfc.nasa.gov/). Both layers were available at a 30 arc-sec resolution (approximately 1 km). EVI is calculated by combining the near infrared and red reflectance properties of vegetation to discriminate vegetation from other objects, and by reducing the influence of soil and atmospheric noise in the vegetation index, generating a more reliable vegetation productivity index as compared for example to NDVI (Sims et al. 2006). Since EVI was available monthly, we downloaded 1 month representing each austral season in 2004 (January, April, July, and October) and calculated the mean EVI values for each grid cell. We chose 2004 because data on land use were available for the same year, and this was an average year (no major heat wave or other extreme environmental event recorded). EVI was upscaled to 10-km resolution by using focal mean statistics.

Land use data were downloaded from the same source and processed using ArcGIS 9.2 to calculate the percent of each land use category in each 10 × 10 km grid cell: in each 10-km grid cell, we calculated the percent covered by evergreen, deciduous, and mixed forests, closed and open shrubland, woody and nonwoody savannas, grasslands, croplands, mixed crops and natural vegetation, urban areas, and barren or scarcely vegetated soil. The mean and range of all environmental variables considered are shown in Appendix 1.

### Statistical analysis

#### Characterization of environmental occupancy

All statistical analyses were carried out using R v2.8.1 (R Development Core Team 2005). In a first step, we needed to compare the actual occurrences of *Rhabdomys* and all lineages with the overall available information on the environment in the study area. Since the number of grid cells without any records of *Rhabdomys* was much larger (roughly 19,500 grid cells) than the number of occupied cells in the study area (*n*= 520), doing so with the full dataset would have artificially weighted absence records. Furthermore, those absences were both the result of no recorded individuals in a grid cell and the lack of visits to these areas. While different strategies to choose these pseudoabsences have been proposed in the literature (Phillips et al. 2009; VanDerWal et al. 2009; Warton and Shepherd 2010), we had to choose a pragmatic approach. Furthermore, species prevalence at around 50% has often been shown to produce better results in statistical modeling (Fielding and Bell 1997; Reese et al. 2005). We therefore generated a dataset that contained all grid cells with records of *Rhabdomys* (*n*= 520), plus an equal number of randomly chosen grid cells with no records of *Rhabdomys*, and used this for the remainder of the statistical analysis. The dataset for the following statistical analyses therefore consisted of these 1040 grid cells, half of which contain presence records for the genus. The added pseudoabsences can be seen more appropriately as background data that will sample all the available environmental space rather than just the sites where *Rhabdomys* have been recorded (Phillips et al. 2009).

To characterize the environmental occupancy of each lineage, we carried out an Outlying Mean Index analysis (OMI) (Doledec et al. 2000). This is a multivariate analysis designed to study niche separation along environmental gradients and that has the advantage of considering linear as well as unimodal relationships between species occurrence and environmental gradients (Doledec et al. 2000; Thuiller et al. 2004). The environmental gradients are summarized through an ordination technique that looks for the optimal combination of environmental variables that separate species occurrences. We therefore carried out the OMI analysis in order to find the environmental gradients that best separate lineages in terms of their occurrence (Doledec et al. 2000), and we calculated the niche position (mean environmental conditions of occurrence) and niche breadth (variance around the niche position) of each lineage along each of the ordination axes (Thuiller et al. 2004). In order to test whether niche position and breadth for each lineage were significantly different from random, we randomly shuffled lineage occurrences within the environment and calculated the same statistics 1000 times. If the observed value of niche position and niche breadth was within the 2.5% lower or upper quantiles of distribution of the randomizations (bootstrap two-tailed test), the observed value was considered as significantly different from random (Thuiller et al. 2004). We repeated the OMI analysis 10 times using different random samples to represent the background environmental gradients, always finding consistent results. For simplicity, we only show results from one of those analyses.

### Projecting potential distributions for each taxon

Since the number of occurrences in each taxon is fairly limited, we could not use conventional statistical methods to project geographically the potential distributions of each clade. Instead, we used MAXENT (Phillips et al. 2006), a maximum entropy algorithm that has been shown to perform fairly well even with a limited number of occurrences, and that can use presence-only data to project species potential distributions (Elith et al. 2006). We therefore used this algorithm, along with the same environmental variables described above, in order to obtain potential distribution maps for *Rhabdomys* as a whole, as well as for the two species separately (*R. pumilio* and *R. dilectus*) and for each one of the proposed subspecies: *R. d. chakae* and *R. d. dilectus*. MAXENT results are presented as a relative suitability index, where a higher value indicates a higher probability of occurrence of the species. To select a threshold above which the species will be considered as present, we used two different methods: the Kappa maximization method and the maximization of the difference between sensitivity and specificity, also known as Maximum Difference Threshold (MDT) (Liu et al. 2005; Jimenez-Valverde and Lobo 2007). These statistics were calculated on the random subset described above for the statistical analysis (*n*= 1040, including all presences of *Rhabdomys* and a random subsample of absences where *Rhabdomys* prevalence was forced to be 50%). Performance of each model and each strategy are given in terms of sensitivity (success rate in predicting presences), specificity (success rate in predicting absences), and in terms of the Area Under the receiving operator Curve (AUC) that is independent of the threshold chosen and considers both presence and absence classification success rates (Fielding and Bell 1997).

## Results

### Characterization of environmental occupancy

The first two ordination axes, hereafter referred to as OMI1 and OMI2 respectively, explained 90 % of the niche separation between taxa (Table 2 and Appendix 2). These ordination axes correspond to a combination of environmental variables that maximize the observed difference in the lineages’ occurrences. The first axis represents the main environmental gradient explaining differentiation within *Rhabdomys* going from warm and dry habitats with low vegetation productivity, to colder and more humid habitats with high vegetation productivity although topographically heterogeneous (Table 2). The other ordination axis explained a lower proportion of the niche differentiation between lineages but still >15% (Table 2). OMI2 represents a gradient from less to more seasonal environments with a wet colder season and a dry warmer season, and from grassland to open shrubland (Table 2 and Appendix 2).

**Table 2:** Outlying Marginality Index (OMI) axes and their interpretation (details in Appendix 3). The general relationship to the environmental variables used in the analysis are provided by category, then an overall interpretation of each axis is given, as well as the corresponding % variance explained by each axis.

	OMI1	OMI2
Temperature	Strong negative relationship to temperature.	Negative relationship to temperature during the wettest season, but positive relationship to temperature during the driest quarter.
Precipitation	Strong positive relationship to precipitation throughout the year, and negative relationship to precipitation seasonality.	Negative relationship to precipitation during the warmest and wettest seasons and precipitation seasonality, but positive relationship to precipitation during the driest and coldest seasons.
Altitude	Strong positive relationship with altitudinal heterogeneity.	Strong positive relationship to altitudinal heterogeneity.
Land use	Positive relationship to % urban and mixed crop-natural vegetation, forests and savannas, and vegetation productivity index; negative relationships to open shrubland and barren ground.	Strong negative relationships to % grassland, negative relation to % urban, and positive to % open shrubland.
Interpretation for high values on each axis.	Cold and humid environments, habitats topographically heterogeneous, dominated by mixed crop-natural vegetation with little open habitat and high productivity.	Seasonal environments, with a wet colder season and a dry warmer season, often dominated by open shrublands.
% Explained	74.6%	15.6%

**Figure 3:**
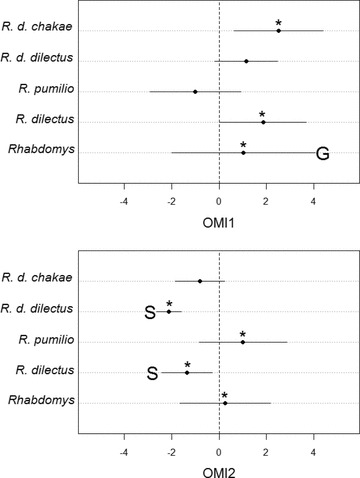
Niche position and breadth along the first three environmental Outlying Mean Index (OMI) axes for each *Rhabdomys* lineage. Values near 0 indicate environmental conditions near the available average conditions for that environmental gradient, whereas values far from this origin indicate marginal environments, that is, environments that are far from the environmental mean conditions. The asterisk indicates that the niche position is significantly different than that expected by random placement of the same number of occurrences (bootstrap two-tailed test, *P* < 0.05). S indicates that the niche breadth is significantly narrower than expected by chance (S = specialist); G indicates that the taxon shows a significantly wider niche breath than expected by chance (G = generalist) (bootstrap two-tailed test, *P* < 0.05).

*Rhabdomys* as a genus shows a significantly wider niche breadth than expected by chance in the first OMI axis, and its niche is significantly different from 0 (i.e., different from mean available environmental conditions) in both axes (Fig. 3). Considering each species separately reveals different occupancy requirements and patterns of specialization within the genus. *Rhabdomys dilectus* shows significant positive values in niche position along OMI1 and both significant negative position and a narrower niche breath than expected by chance in OMI2 (Fig. 3). The two *R. dilectus* subspecies show only small differences: *R. d. chakae,* but not *R. d. dilectus,* shows a significantly positive niche position along OMI1, while *R. d. dilectus,* but not *R. d. chakae*, shows a significantly positive position and narrow niche breadth along OMI2. *Rhabdomys pumilio* niche position along OMI1 is negative but not significantly different from 0, while its position on OMI2 is significantly positive (Fig. 3).

Taken together these patterns support the idea that *R. dilectus* occurs in wet and relatively cold environments with a hot rainy season and a drier colder season supporting mixed crop-natural vegetation and grassland with little open habitat and high productivity (Table 2 and Fig. 3). On the other hand *R. d. chakae* occurs in more humid and colder environments than *R. d. dilectus*, the latter being found in warmer and more stable environments characterized by grassland-type vegetation. In contrast to *R. dilectus*, environments occupied by *R. pumilio* appear to be more seasonal with a dry warm season and a wet colder season, the vegetation being scarcer and more patchily distributed than where *R. dilectus* occurs.

### Projecting potential distributions for each taxon

**Figure 4:**
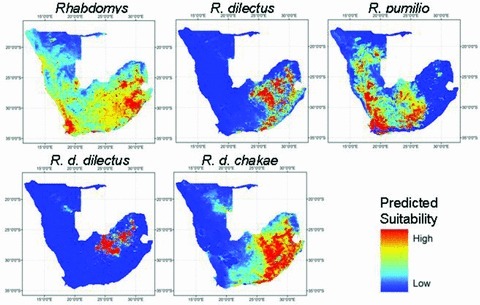
Predicted suitability maps using MAXENT for all lineages of *Rhabdomys*.

**Figure 5:**
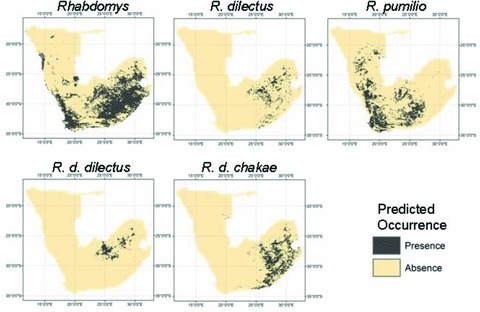
Predicted distribution maps using MAXENT for all lineages of *Rhabdomys* based on maximum difference threshold (MDT) threshold (see Methods for details and Appendix 3 for distribution maps based on KMT thresholds).

**Figure 6:**
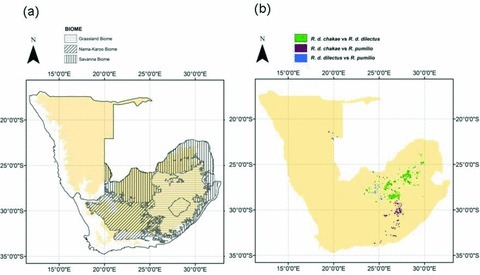
Predicted contact zones between lineages. Panel (a) shows the major biomes at the interface of contact zones in South Africa, as well as the 900-m contour (yellow background) that constitutes an approximate limit for the escarpment area; panel (b) shows the actual predicted contact zones. These were drawn based on areas of overlap between the different lineages using MAXENT projections and a maximum difference threshold (MDT) to predict lineages occurrences (see Methods).

All projections show fairly high predictive success rates, with AUC often >0.9, the only exception being the projections for *Rhabdomys* as a genus (Table 3). Suitability maps produced using MAXENT result in differential distributions for all taxa (Fig. 4). Using Kappa Maximizing Threshold (KMT) to determine the threshold for presence–absence predictions usually results in an excellent specificity (i.e., little error rate in absence predictions) but sometimes a poor sensitivity (i.e., a poor error rate in presence predictions) (Table 3). We therefore present occurrence predictions based on MDT (Fig. 5), which resulted in a more balanced sensitivity and specificity (Table 3). Occurrence maps based on the KMT strategy are provided in Appendix 3. Under our prediction, *R. pumilio* presents the largest range as it occurs in the western (drier) part of the distribution range, but also penetrates further into the central part of southern Africa. While *R. dilectus* range appears to cover the eastern (wettest) part of southern Africa, with *R. d. chakae* occupying a larger area than *R. d. dilectus*, the latter being restricted to the northeastern part of our study area. Zones of overlap are predicted between the three taxa (Fig. 6).

**Table 3:** Summary of model performance predicting species occurrences of *Rhabdomys*. Results are shown according to the threshold used to determine species presence or absence from the suitability predicted by MAXENT. AUC, performance index; KMT, kappa maximizing threshold; MDT, maximum difference threshold. Notice that while AUC is threshold independent, sensitivity (presence prediction success rate) and specificity (absence prediction success rate) depend on the threshold chosen to predict occurrence from the continuous suitability index.

		Threshold values		KMT		MDT	
Taxon	AUC	KMT	MDT	Sensitivity	Specificity	Sensitivity	Specificity
*Rhabdomys*	0.83	0.34	0.39	0.84	0.66	0.73	0.74
*Rhabdomys dilectus*	0.97	0.84	0.38	0.43	0.99	0.95	0.93
*Rhabdomys pumilio*	0.93	0.70	0.41	0.53	0.98	0.83	0.84
*Rhabdomys dilectus chakae*	0.91	0.78	0.57	0.17	0.99	0.92	0.86
*Rhabdomys dilectus dilectus*	0.99	0.59	0.31	0.80	0.98	1	0.95

## Discussion

Museum collections provide a plethora of species occurrence data resulting from heterogeneous and often unknown sampling schemes. In parallel, accurate environmental data for large geographical areas are now available, providing powerful opportunities for zoologists aiming to gain a broader view on the distribution and ecological characteristics of their model taxa (Graham et al. 2004; Elith and Leathwick 2007). The present study has used these tools to assess environmental predictors of *Rhabdomys* distribution, to test whether environmental niche differentiation has occurred in a genus long thought to be monospecific, and to predict where contact zones between its different taxa may occur. Patterns of environmental occupancy of *Rhabdomys*, as revealed in our study, indicate that historical considerations of the genus as a generalist (e.g., Skinner and Chimimba 2005) most probably result from this rodent being a complex of taxa with different ecological characteristics. Furthermore, the two prerequisites for identifying secondary contact zones are (1) knowledge about the taxonomic identity of the taxa, and (2) characterization of the distribution range of each taxonomic entity. While Rambau et al. (2003) and Castiglia et al. (2011) provided important taxonomic clarifications for *Rhabdomys*, we provided here a thorough environmental characterization of the genus occupancy range that has not been attempted before, except at the much smaller scale of the Free State Province (Ganem et al. In Press). We therefore discuss these potential contact zones in more detail below.

Our results reveal at least two interesting aspects of the environmental occupancy patterns of *Rhabdomys*. First, different taxa occupy different portions in the available environmental gradients. Only two environmental axes related to temperature and precipitation, and environmental variability gradients (Table 2) explained a large portion of environmental differentiation between *Rhabdomys* taxa. These environmental variables have played a major role in the radiation of the genus (Rambau et al. 2003), and mirror previous suggestions that major biomes such as savannah and grasslands, which are greatly dependent on precipitation and temperature regimes, had major influences on the diversification of South Africa through their repeated expansion and contraction during the Miocene and Pliocene (e.g., Daniels et al. 2006, 2009; Willows-Munro and Matthee 2011). This is also consistent with the Pliocene being a period of multiple episodes of aridification, resulting in landscape discontinuities over southern Africa (DeMenocal 2004), and which effected species diversification (Frey 1992; Lecompte et al. 2008; Mirol et al. 2010; Russo et al. 2010). Second, environmental divergence within *Rhabdomys* parallels phylogeny as the two species appear to be more distinct than the two subspecies. *Rhabdomys pumilio* occupies warmer, drier, and more seasonal environments and appears to be able to thrive in habitats with less cover and lower productivity (e.g., the semidesert succulent Karoo) than *R. dilectus*. The two *R. dilectus* are always found in the same side of the environmental axes. Our results indicate that habitats where *R. d. dilectus* is found are characterized by grassland-type vegetation and are warmer and less seasonal (e.g., Kwazulu-Natal midlands) than those of *R.d. chakae* that is characterised by both grassland and mixed crop-natural vegetation with high productivity index (e.g., northern parts of South Africa, extending into Zimbabwe).

Fossil and paleoclimate information may help us picture the original habitat of *Rhabdomys* ancestors. The earliest known *Rhabdomys* fossil was found in the southwestern coast of South Africa about 5 million years ago (Denys 1999 and references therein), suggesting that *Rhabdomys* ancestors thrived in a region where populations of *R. pumilio* still occur today. Further, isotopic (δ^13^C) investigations in the fossil site of Langebaanweg (Western Cape, SA) indicated that the region was probably a winter rainfall area characterized by a C3-type vegetation at that time (Segalen et al. 2007), as opposed to C4-grassland-type vegetation in a mosaic landscape of woodland patches described at an earlier period (4.0–3.5 Ma) in the Free State (de Ruiter et al. 2010). The ancestral habitat of *Rhabdomys* could therefore resemble that of the western range of the actual *R. pumilio*: a winter-fall region (WFR) mainly characterized by arid open-shrubland-type vegetation. *Rhabdomys* would have colonised the central and eastern regions only secondarily (unlike suggested by Rambau et al. 2003), possibly at a period when these regions had a different climate. Divergence within *Rhabdomys* occurred at dates similar to other rodents, such as the Namaqua rock mouse (Russo et al. 2010), and some invertebrates (Daniels et al. 2006), supporting the links to climate cycles and consequent biome expansions and retractions leading to population fragmentations and differentiation.

The distribution maps generated in this study reveal several potential contact zones and potential areas of localized sympatry along the sharp altitudinal gradient found in the southern tip of South Africa and the escarpment (e.g., the Cape Fynbos), in the grassland biome, as well as near its limits with the Nama Karoo and the savanna biome in the central part of South Africa (e.g., the Free State Province) (Fig. 6). As expected, given their relatively similar environmental characteristics, contact zones between the two *R. dilectus* subspecies, are predicted to cover large areas, mainly within the grassland biome, with the only visible barrier being large urban areas of the Gauteng province, for example. However, several sites within this potential contact zone were visited and trapped and yielded only one or the other subspecies to date (Pillay et al. 2006 and subsequent unpubl. ms.). Further studies are needed to confirm the absence of contact zones and disentangle the factors involved. Competition may be an important ecological driver of species range boundaries in these areas where no obvious dispersal or habitat barrier exist and where the environment seems suitable for two subspecies but only one of them has been observed. Based on its pattern of mitochondrial divergence, Rambau et al. (2003) proposed that this subspecies show signs of recent expansion. It would therefore be interesting to use the contact zones map produced here to investigate whether these areas are actually occupied by a single subspecies, possibly *R. d. chakae*, which would suggest that competitive replacement may occur between the two subspecies.

Potential contacts between *R. pumilio* and the two *R. dilectus* subspecies occur mainly along a north to south axis near longitude 27°E, with predictions of contact between the three taxa in the north of South Africa in edge regions of both the savannah and grassland biomes (Fig. 6). The contact zone between *R. d. chakae* and *R. pumilio* is also predicted to occur partly within the grassland biome, although in a region of important altitudinal heterogeneity close to the western part of the Drakensberg mountain range (Free State Province), the southern part of the escarpment, and down to the coastal area of Eastern Cape. In the southern part of this potential contact zone, the Zuurberg Mountains could be separating these forms, with an additional change in vegetation from Nama Karoo to bushveld thicket. For the contact zone between *R. d. dilectus* and *R. pumilio*, there is no obvious physical barrier (as *R. pumilio* seems to have crossed the Orange River). The maps shown here predict this contact zone to occur within the grassland biome, which was confirmed by recent field work (Ganem et al. In Press).

Contact zones constitute candidate regions where future studies could investigate the extent of ecological divergence between the taxa (Fig. 6) (Rundle and Nosil 2005; Rissler and Smith 2010). Candidate areas of contact between the different lineages of *Rhabdomys* pinpointed in our study will allow effective targeting of field sites for further studies, allowing study of the current interplay between evolutionary and ecological mechanisms underlying speciation. For example, genetic divergence within *Rhabdomys* is also associated with behavioral divergence: high levels of aggression between lineages (Pillay 2000), divergence of the mate recognition system (Pillay et al. 2006), and regional differences in social organisation and reproduction (Schradin and Pillay, 2005b; Krug 2007). Differences in the behavior and ecology of the two main lineages described by Rambau et al. (2003) seem to be related to differences in population density, food availability (Schradin and Pillay 2005b; Schradin et al. 2010), and low nocturnal temperatures, resulting in the need for huddling in the desert (Scantlebury et al. 2006), factors that could all be influenced by prevailing climatic conditions and expected climate change. Overall, climate-dependent flexibility of behavior could have been a key characteristic in the radiation of this genus (Schradin et al. 2011). Therefore, the study of these social and behavioral characteristics within and among the different lineages throughout their distribution range may help us understand the potential effects of future rapid environmental and climate change on these populations.

The maps of potential distributions for each taxon proposed here (Figs. 4 and 5; Appendix 3) can only be viewed as spatially explicit hypotheses of the distribution of each taxon. Indeed, the modeling effort carried out here presents some shortcomings linked to the limited number of occurrences used in the modeling process (Table 1). A minimum number of 20–30 species occurrences has generally been recommended for use with MAXENT (Franklin 2009) and 50–70 occurrences are more generally recommended for species distribution modeling (Stockwell and Peterson 2002; Wisz et al. 2008). However, MAXENT has been tested previously with very low occurrence numbers comparable to those used here, generating reasonably good distribution maps (Elith et al. 2006; Hernandez et al. 2006; Graham et al. 2010), and has generally been accepted as an appropriate tool for modeling species distributions when presence-only data are available (Elith et al. 2006). The lowest number of occurrences used here corresponds to *R. d. dilectus*, and is within the range of occurrence records tested in Hernandez et al. (2006), suggesting that even under these circumstances, our predictions can be useful hypotheses of the distribution of the taxa.

Overall, our study pinpoints the urgent need to clarify the taxonomic status and distribution regarding different African taxonomic groups. Understanding the interplay between environmental and ecological factors shaping large scale diversity patterns requires a joint effort of this kind and is fundamental to providing realistic scenarios of biodiversity threats under climate change. We have provided here some hypotheses regarding the diversification of one rodent genus, *Rhabdomys*. However, our hypotheses could be validated if evidence from a diversity of taxonomic groups in the study region shows similar patterns. The identification of potential contact zones between the different taxa will also allow us to target future field and experimental work in order to tease apart the roles of ecological forces, such as competition, at interplay with evolutionary mechanisms, such as gene flow and hybridization between recently differentiated clades, in shaping species distribution boundaries, and in the maintenance of biodiversity.

## Appendix 1

Summary of data used for the statistical analysis.Table A1 provides a summary of the various sources used to compile andgeoreference *Rhabdomys* records, while Table A2 provides a summary of environmental conditions in the study regionincluding all environmental variables used as well as their mean and ranges.

**Table A1:** Summary of various sources used to georeference records of *Rhabdomys* sp.

Origin of data	Number of occurrences
Transvaal Natural History Museum, Pretoria, SA	345
Iziko Museums of Cape Town, SA	243
Durban Natural Science Museum, Durban, SA	993
McGregor Muséum, Kimberley, SA	188
Natural History Museum, Windhoek, Namibia	572
National Museum, Bloemfontein, SA	1968
MNHN, National Natural History Museum, Paris, France	18
ICT Africa Museum, Belgium. http://projects.biodiversity.be	42
National Museum of Natural History, Washington, USA	1567
Rambau et al. (2003)	18*
Ganem et al. (In Press)	102*

* Genotyped samples.

**Table A2:** Detailed description of environmental variables used in the statistical analysis for the distribution of *Rhabdomys*. Climatic variables were downloaded from the freely available database Worldclim (http://www.worldclim.org). Climatic variable descriptions can be found in (Hijmans et al. 2005), and correspond to interpolated minimum, maximum, and mean values of temperature and precipitation gathered between 1950 and 2000. Notice that temperature is represented as temperature in °C × 10, temperature seasonality corresponds to standard deviation of monthly data × 10, isothermality corresponds to the ratio between diurnal and annual temperature range, and precipitation seasonality corresponds to the coefficient of variation of the monthly data. A vegetation productivity index, the Enhanced Vegetation Index (EVI), as well as land use were downloaded from Modis Terra. Land use categories were further transformed into percentage of each category within each 10 × 10 km grid in our analysis.

Variable	Mean	Standard deviation	Minimum value	Maximum value
Annual mean temperature	174.86	28.20	51.00	229.00
Mean diurnal range	148.97	22.32	66.00	189.00
Isothermality	56.05	4.32	45.00	72.00
Temperature seasonality	4040.70	1014.81	1473.00	6331.00
Max temperature of warmest month	296.54	37.06	145.00	387.00
Min temperature of coldest month	30.96	35.37	–58.00	123.00
Temperature annual range	265.58	47.65	117.00	355.00
Mean temperature of wettest quarter	205.79	39.38	80.00	269.00
Mean temperature of driest quarter	132.01	42.55	5.00	252.00
Mean temperature of warmest quarter	220.95	28.68	90.00	288.00
Mean temperature of coldest quarter	118.84	32.12	5.00	188.00
Annual precipitation	460.18	265.01	10.00	1159.00
Precipitation of wettest month	83.06	42.79	3.00	228.00
Precipitation of driest month	7.08	7.79	0.00	65.00
Precipitation seasonality	73.11	24.54	10.00	147.00
Precipitation of wettest quarter	226.12	121.66	6.00	629.00
Precipitation of driest quarter	26.23	26.52	0.00	207.00
Precipitation of warmest quarter	188.03	128.86	3.00	629.00
Precipitation of coldest quarter	39.27	59.86	0.00	464.00
Altitude	1108.81	494.03	2.00	3231.00
Spatial standard deviation of altitude	410.16	362.28	9.00	1957.00
% Evergreen forest	1.44	8.03	0.00	94.00
% Deciduous forest	1.28	3.94	0.00	34.00
% Mixed	0.06	0.36	0.00	4.00
% Closed shrubland	0.44	1.50	0.00	24.00
% Open shrubland	47.60	38.39	0.00	100.00
% Woody savanna	4.72	8.36	0.00	65.00
% Grasslands	11.97	16.30	0.00	77.00
% Crops	1.84	8.29	0.00	100.00
% Urban	2.45	12.39	0.00	100.00
% Mixture crop-natural vegetation	0.75	2.95	0.00	33.00
% Barren soil	8.68	24.11	0.00	100.00
Mean EVI	1829.31	853.99	–3000.00	4816.50

## Appendix 2

Details of Outlying Mean Index (OMI) analysis loadings. Each column represents the coefficients, that is, relative influence, of each variable for each OMI axis.

**Table:**

Variable	OMI1	OMI2
Annual mean temperature	−0.29	−0.06
Mean diurnal range	−0.15	−0.05
Isothermality	−0.10	−0.03
Temperature seasonality	0.00	0.00
Max temperature of warmest month	−0.29	0.00
Min temperature of coldest month	−0.20	0.04
Temperature annual range	−0.08	−0.03
Mean temperature of wettest quarter	−0.20	−0.15
Mean temperature of driest quarter	−0.20	0.10
Mean temperature of warmest quarter	−0.28	−0.03
Mean temperature of coldest quarter	−0.25	−0.04
Annual precipitation	0.32	−0.07
Precipitation of wettest month	0.22	−0.12
Precipitation of driest month	0.26	0.09
Precipitation seasonality	−0.24	−0.11
Precipitation of wettest quarter	0.23	−0.11
Precipitation of driest quarter	0.27	0.09
Precipitation of warmest quarter	0.29	−0.13
Precipitation of coldest quarter	0.15	0.15
Altitude	0.10	−0.10
Spatial standard deviation of altitude	0.20	0.17
% Evergreen forest	0.11	0.06
% Deciduous forest	0.10	0.03
% Mixed	0.07	0.03
% Closed shrubland	0.06	0.05
% Open shrubland	−0.19	0.12
% Woody savanna	0.09	−0.04
% Grasslands	0.07	−0.18
% Crops	0.10	0.07
% Urban	0.21	−0.12
% Mixture crop-natural vegetation	0.17	−0.03
% Barren soil	−0.11	0.04
Mean EVI	0.21	−0.04

## Appendix 3

Distribution maps generated using the maximum kappa (KMT) threshold criteria for the different lineages of *Rhabdomys* (see Methods).
